# Sickness Absence following Coronary Revascularisation. A National Study of Women and Men of Working Age in Sweden 1994–2006

**DOI:** 10.1371/journal.pone.0040952

**Published:** 2012-07-24

**Authors:** Margaretha Voss, Torbjörn Ivert, Kenneth Pehrsson, Niklas Hammar, Kristina Alexanderson, Tage Nilsson, Marjan Vaez

**Affiliations:** 1 Division of Insurance Medicine, Department of Clinical Neuroscience, Karolinska Institutet, Stockholm, Sweden; 2 Department of Cardiothoracic Surgery and Anesthesiology, Karolinska University Hospital, Stockholm, Sweden; 3 Department of Cardiology, Karolinska University Hospital, Stockholm, Sweden; 4 AstraZeneca R&D, Södertälje, Sweden; 5 Institute of Environmental Medicine, Karolinska Institutet, Stockholm, Sweden; 6 PCI-unit, Central Hospital, Karlstad, Sweden; John Hopkins Bloomberg School of Public Health, United States of America

## Abstract

**Background:**

Evidence based and gender specific knowledge about sickness absence following coronary revascularisation is lacking. The objective was to investigate sickness absence after a first coronary artery bypass grafting (CABG) or percutaneous coronary intervention (PCI) among women and men in a national Swedish study.

**Materials and Methods:**

All patients 30–63 years of age, who underwent a first CABG (n = 22,985, 16% women) or PCI (40,891, 22% women) in Sweden between 1994 and 2006 were included. Information on sickness absence, co-morbidity, and other patient characteristics was obtained from national registers. Long-term sickness absence (LTSA) was defined as >180 and >90 sick-leave days in the first sick-leave spell following CABG and PCI, respectively. Prevalence ratio (PR) and 95% confidence interval (CI) of LTSA were calculated.

**Findings:**

LTSA followed the interventions in 41% and 36% for CABG and PCI patients, respectively. Women had more often LTSA compared with men, (CABG PR = 1.23: 95% CI 1.19–1.28 and PCI PR = 1.19; 95% CI 1.16–1.23). A history of sickness absence the year before the intervention increased the risk for LTSA after the intervention in both genders. Among women, older age, or being self employed or unemployed was associated with a lower risk for LTSA. Among men previous cardiovascular disease, diabetes and low socio-economic position increased the risk. During the observation period, there was no change in sickness absence rates among PCI patients but an increase among CABG patients adjusting for patient characteristics.

**Conclusion:**

This national study covering a 13-year period shows that long-term sickness absence following coronary revascularisation is common in Sweden, especially among women, and is associated with socio-economic position, co-morbidity, and sickness absence during the year before the intervention. Gender specific scientific knowledge about use and effects of sickness absence following coronary revascularisation is warranted for the patients, the treating physicians, the healthcare sector, and the society.

## Introduction

Coronary revascularisation by coronary artery bypass grafting (CABG) and percutaneous coronary intervention (PCI) are established treatments of acute as well as stable coronary heart disease. CABG and PCI improve quality of life and in subgroups of patients mortality is reduced [1]–[2]. Although sickness absence following these interventions is almost always the rule, the scientific knowledge about optimal time for this is lacking. So far, there are only few studies of sickness absence after CABG or PCI considering how common such interventions are [3]–[11].

**Figure 1:**
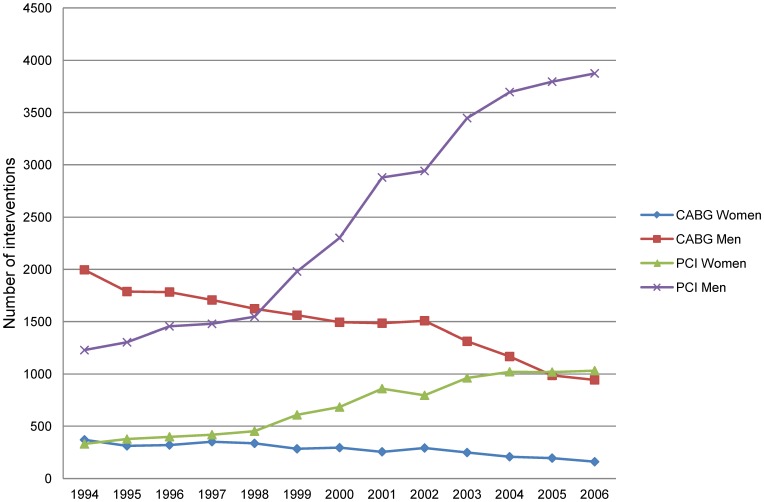
First time CABG and PCI in Sweden in 1994–2006.

In Sweden, today about 10 000 individuals below age 65 undergo CABG or PCI each year [12]. During the last decades, treatment with PCI has increased continuously in Sweden and has replaced CABG in most patients with single and double vessel coronary artery disease [1]–[2]. There have been substantial changes in the patient population undergoing CABG as well as PCI with an increasing proportion of patients with more severe coronary artery pathology, co-morbidity, and cardiovascular risk factors, and gender differences in these factors are well described [13]–[16].

Among patients working, most return to work after PCI and CABG; faster after PCI [3], [8], [11], [13]. Only few studies on sickness absence after coronary revascularisation have been performed in Sweden and none of them presented gender specific results [5]–[6]. A randomised study (The FRISC II-study) found a mean number of 102 sick-leave days within the year following PCI or CABG, among working subjects [6]. Compared to other countries, patients in Sweden have had 2–5 times longer sick-leave spells after coronary revascularisation [6]. Evidence based knowledge for this practice is lacking [9].

There is reason to believe that sickness absence following CABG and PCI differs between women and men. Women are in general older than men at the onset of cardiovascular disease and at the time of need for coronary revascularisation [16]. In addition, women undergoing CABG have more co-morbidity, are smaller and thus have smaller coronary arteries than men which may be associated with a worse prognosis after CABG [13]. Studies have furthermore suggested that women with stable angina are often sub optimally treated, and if less than 70 years, have a poorer survival [14]–[15]. Finally, women have higher levels of sickness absence in general compared with men and there might be gender differences in mechanisms behind sickness absence [17]–[18].

The aim of this study was to investigate sickness absence after a first coronary revascularisation by CABG or PCI among women and men of working age in Sweden during the period 1994–2006, taking patient characteristics, including morbidity and history of sickness absence into account.

**Table 1:** Patient characteristics.

		CABG																PCI													
		Women							Men									Women									Men				
		*n = 3627*							*n = 19358*									*n = 8961*									*n = 31930*				
		*No.*	*%*						*No.*			*%*						*No.*				*%*					*No.*			*%*	
*Age at intervention (years)*																															
30–49		510		14.1				2559				13.2					1613					18.0				6547					20.5
50–54		645		17.8				3933				20.3					1850					20.6				7037					22.0
55–59		1144		31.5				6491				33.5					2729					30.5				10159					31.8
60–63		1328		36.6				6375				32.9					2769					30.9				8187					25.6
*Indication*																															
MI^1^ or acute coronary syndrome		3573			57.0				10619				56.1				5579					62.3						20077			63.0
Stable angina pectoris		1402			39.2				7682				40.5				3115					34.8						10847			34.0
Other		135			3.8				646				3.4				265					3.0						990			3.1
*Hospitalisation 5 years before intervention*																															
Angina pectoris	2563					70.7				13175				68.1				2925			32.6							9112			28.5
Acute myocardial infarction	1272					35.1				7219				37.3				1790			20.0							6823			21.4
Diabetes mellitus^2^	1113					34.9				3883				23.4				1696			21.1							4700			16.5
Heart failure	334					9.2				1406				7.3				294			3.3							920			2.9
Cerebrovascular disease	130					3.6				529				2.7				179			2.0							534			1.7
Peripheral vascular disease	148					4.1				537				2.8				178			2.0							401			1.3
Asthma/obstructive pulmonary dis.	136					3.7				306				1.6				251			2.8							389			1.2
Mental disorders	183					5.0				967				5.0				393			4.4							1360			4.3
Musculoskeletal disorders	375					10.3				1139				5.9				732			8.2							1672			5.2
Other diagnoses^3^	172					4.7				615				3.2				352			3.9							847			2.7
*Socio-economic classification* 4																															
Manual workers	1440					50.8				7471				44.0				3652					49.4					12435			44.4
Assistant non-manual employees	642					22.6				1716				8.9				1529					20.7					2789			10.0
Higher non-manual employees^5^	486					17.1				5229				30.8				1508					20.4					8577			30.6
Self employed + farmers	127					4.5				1633				9.6				320					4.3					2517			9.0
Unclassified	141					5.0				928				5.5				386					5.2					1693			6.0
*Employment status*																															
Employed	1837					50.7				11463				59.2				4257					47.5					16170			50.6
Self employed	96					2.7				1334				6.9				268					3.0					2709			8.5
Unemployed	438					12.1				2548				13.2				1753					19.6					7420			23.2
Others or unknown	1256					34.6				4013				20.7				2683					29.9					5631			17.6
*Disability pension at the time of intervention*																															
>50%	1141						31.5				3423				17.7				2431					27.1				4435			13.9
≤50%	312						8.6				925				4.8				734					8.2				1232			3.9
*Sickness absence * ***the year before the intervention***																															
Patients with sick-leave days	2224					61.3					14076					72.7				4687					52.3				17027		53.3
Patients with >90 sick-leave days	1032					28.5					5319					27.5				1929					21.5				5559		17.4

1 Myocardial infarction.

2 Primarily from the quality registers or hospitalised diabetes mellitus from the Patient register.

3 Cancer, renal failure and transient cerebral ischaemic attacks.

4 Percent of those with a socio-economic classification.

5 Including non-manual employees at intermediate level.

## Methods

### Ethics Statement

The study population was identified through hospital records collected and stored with the consent of the patients. Additional information was collected by linkage of several public national registers. Ethical vetting is always required when using register data in Sweden. The ethical vetting is performed by regional ethical review boards and the risk appraisal associated with the Law on Public Disclosure and Secrecy is done by data owners. The ethical review boards can however waive the requirement to consult the data subjects directly to obtain their informed consent, and will often do so if the research is supported by the ethical review board and the data has already been collected in some other context. According to these standards in Sweden this project has been evaluated and approved by the Regional Ethical Review Board in Stockholm, Sweden.

### Study Population

The study encompasses all individuals (*n* = 63 876), who underwent a first CABG (*n* = 22 985) or PCI (*n* = 40 891) in Sweden during the 13-year period 1994–2006, were 30–63 years of age at the time of intervention, and did not die or have a re-intervention within 30 days after the index intervention. The mean age at the time of CABG was 56.3 and 56.1 years, among women and men, respectively, and among those who underwent PCI 55.2 and 54.6 years, respectively.

The patients were recruited from two national registers; the Swedish Heart Surgery Registry and the Swedish Coronary Angiography and Angioplasty Registry (SCAAR) [12]. These registers include all patients who had undergone CABG and PCIs performed in Sweden during the inclusion period, and our cohort represents 35% and 31% of all CABG and PCI patients reported during the period 1994–2006. Data included information about indication, date of intervention, type of procedure, and co-morbidity.

**Table 2:** Sick-leave days in the first sick-leave spell following coronary revascularisation.

	CABG				PCI			
	Women		Men		Women		Men	
	*n = 2426*		*n = 15470*		*n = 6402*		*n = 26770*	
	*No.*	*%*	*No.*	*%*	*No.*	*%*	*No.*	*%*
≥1 sick-leave day	2110	87.0	13869	89.7	4847	75.7	19248	71.9
>90 sick-leave days	1843	76.0	10716	69.3	2937	45.9	9161	34.2
>180 sick-leave days	1271	52.4	6041	39.0	2203	34.4	6200	23.2
≥365 sick-leave days	825	34.0	3717	24.0	1512	23.6	3914	14.6
Mean number	241.5		198.5		188.1		147.5	
Median number	253.5		151.0		146.0		83.0	
Median number (weighted)^1^	200.0		132.0		71.0		47.0	

1 Those with no data on sick-leave benefits following the intervention were coded as having had 14 sick-leave days, in order to not underestimate the number of sick-leave days.

**Figure 2:**
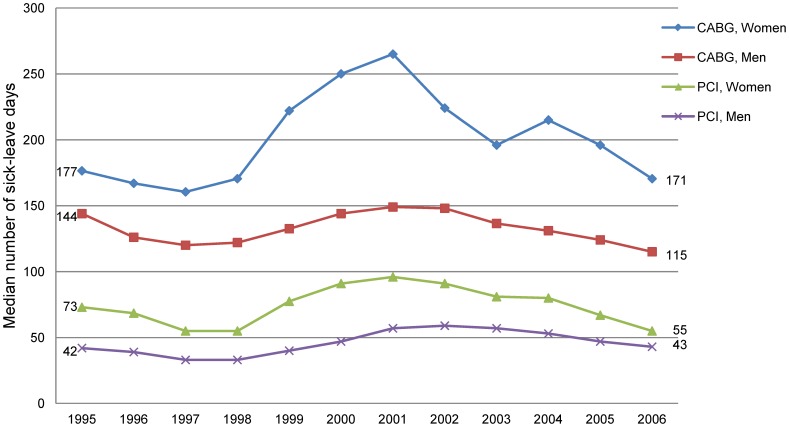
Median number of sick-leave days in the sick-leave spell following CABG and PCI in 1994–2006.

**Figure 3:**
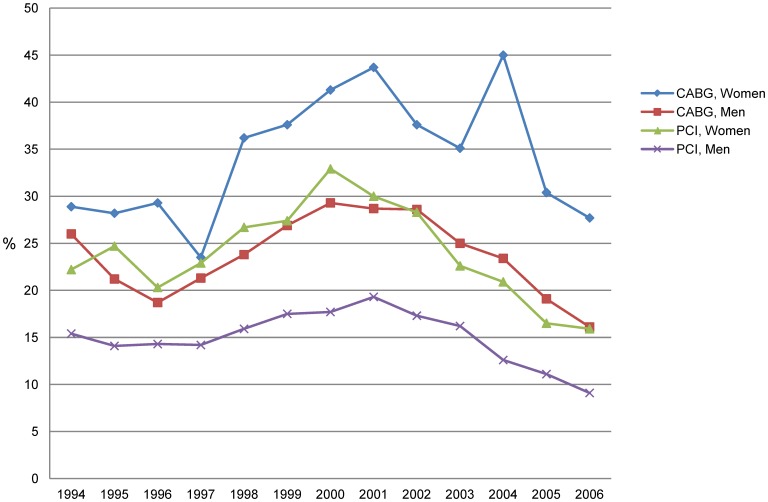
Proportion of patients with at least 365 sick-leave days in the sick-leave spell following CABG and PCI in 1994–2006.

### Linkage to National Registers

Information from national registers was linked to the patients using the unique Swedish personal identification number. Information about sickness absence, disability pension, co-morbidity, mortality, socio-demographic, and socio economic situation was obtained from the Social Insurance Agency (MiDAS data base), the Register of Information and Knowledge about Swedish Heart Intensive Care Admissions (RIKS-HIA), the Board of Health and Welfare (National Patient Register and Cause of Death Register), and Statistics Sweden (LISA register), respectively.

### Patient Characteristics

The following data on patient characteristics were used:

A *history of hospitalisation* for cardiovascular or other major conditions within five years before the intervention was obtained from the Patient Register. The following diagnoses according to the International Classification of diseases, ICD 9 (1987–1996) and ICD 10 (from 1997) were used: acute myocardial infarction, angina pectoris, heart failure, cerebrovascular disease, peripheral artery disease, asthma or chronic obstructive pulmonary disease, diabetes mellitus, transient cerebral ischeamic attacks, renal failure, cancer, mental disorders, and musculoskeletal disorders.

Based on the Population and Housing Census from 1990, the following *socioeconomic* classification was used: manual workers, assistant non-manual employees, higher non-manual employees (including non-manual employees at intermediate level), self employed, and farmers.

*Employment status* at the time of intervention (employed, self employed, or unemployed) was in general based on information from MiDAS. For patients with no sickness absence the employment status was based on data from LISA for the year of the intervention.

**Table 3:** Prevalence ratio of a sick- leave spell >180 days following CABG for *all patients* and for *patients with ≤90 sick-leave days* immediately before the intervention.

CABG	All patients		Patients with ≤90 sick-leave days		
	Women	Men	Women	Men	
	*n = 2426*	*n = 15470*	*n = 1249*	*n = 9577*	
	*PR (95% CI)* 1	*PR (95% CI)* 1	*PR (95% CI)* 1	*PR (95% CI)* 1	
*Age groups (years)*					
30–49	1	1	1	1	
50–54	0.98 (0.89–1.07)	1.02 (0.96–1.08)	1.01 (0.85–1.20)	1.02 (0.93–1.12)	
55–59	0.92 (0.84–1.00)*	1.06 (1.00–1.12)*	1.02 (0.87–1.20)	1.07 (0.97–1.16)	
60–63	**0.78 (0.70–0.86)** ***	1.03 (0.97–1.09)	0.89 (0.75–1.06)	1.07(0.97–1.17)	
Women *vs* Men	**1.23 (1.19–1.28)** ***		**1.50 (1.40–1.60)** ***		
*Indication*					
MI^2^ or acute coronary syndrome					
*vs* stable angina	1.02 (0.95–1.09)	**1.04 (1.01–1.08)** *	1.08 (0.95–1.21)	**1.26 (1.19–1.35)** ***	
Diabetes mellitus	1.05 (0.98–1.13)	**1.10 (1.06–1.15)** ***	1.10 (0.96–1.26)	**1.17 (1.09–1.26)** ***	
*Hospitalisation 5 years before the intervention*					
Cardiovascular disease^3^	1.03 (0.97–1.10)	**1.04 (1.01–1.08)** *	1.03 (0.91–1.16)	**1.18 (1.11–1.25)** ***	
Mental disorders	1.05 (0.92–1.19)	1.02 (0.94–1.11)	1.04 (0.74–1.48)	1.04 (0.88–1.22)	
Musculoskeletal disorders	0.95 (0.84–1.08)	1.07 (1.00–1.15)	1.02 (0.77–1.35)	1.10 (0.95–1.28)	
*Socio-economic classification*					
Higher non-manual employees^4^	1	1	1	1	
Assistant non-manual employees	0.94 (0.85–1.05)	**1.15 (1.07–1.24)** ***	0.86 (0.72–1.03)	**1.23 (1.09–1.38)** ***	
Manual workers	0.97 (0.89–1.06)	**1.34 (1.27–1.41)** ***	1.01 (0.87–1.17)	**1.60 (1.48–1.72)** ***	
*Employment status*					
Employed	1	1	1	1	
Self employed	**0.77 (0.60–0.98)** *	0.98 (0.92–1.05)	**0.62 (0.40–0.96)** *	1.04 (0.93–1.16)	
Unemployed	**0.77 (0.68–0.87)** ***	0.98 (0.93–1.03)	**0.73 (0.59–0.90)** **	0.99 (0.90–1.09)	
*Sick-leave days during the year before CABG*					
>90 *vs* 0–90 sick-leave days^5^	**1.83 (1.70–1.97)** ***	**2.20 (2.12–2.28)** ***	**1.22 (1.03–1.44)** *	**1.36 (1.23–1.51)** ***	

1 Adjusted for age and >90 sick-leave days *in total* during the year before CABG.

2 Myocardial infarction.

3 Acute myocardial infarction, heart failure, cerebrovascular disease and transient cerebral ischeamic attacks.

4 Including non-manual employees at intermediate level.

5 Not adjusted for >90 sick-leave days *in total* during the year before CABG.

* p-value <0.05;

** p-value <0.01;

*** p-value <0.001.

#### Social insurance

In Sweden, sickness-absence insurance covers all inhabitants. During the study period, the *sickness benefit* amounted to at least 80% of lost income up to a certain level and there was no limit to the duration of a sick-leave spell. Sickness benefit could be granted to those who were unable to work due to disease or injury. The first seven days in a sick-leave spell could be self-certified, thereafter, a medical certificate was required. During the study period, the employer usually paid the benefit for employees during the first days of a sick leave spell, thereafter, the benefit was paid from the Social Insurance Agency. Most of the years, employer paid up to day 14. However, between 31 January 1997 and 31 May 1998 that period was 28 days and between 1 July 2003 and 31 December 3, 2004 it was 21 days. For others, e.g. unemployed, the Social Insurance Agency pays all benefits. All have one qualifying day, when no benefit is paid, for self employed this can be 3 or 30 days instead, depending on insurance agreement. All sick-leave days in sick-leave spells with benefits from the National Social Insurance Agency (including the qualifying day and benefit from the employer) are available from MiDAS.

*Disability pension* could be granted to people with long-term work incapacity due to disease or injury and benefits amounted to at least 65% of lost income. Disability pension was categorised as: no disability pension, ≤50%, or >50% of the ordinary working time.

The age for *old-age pension* was 65 years but could be taken earlier.

**Table 4:** Prevalence ratio of a sick-leave spell >90 days following PCI for *all patients* and for *patients with ≤90 sick-leave days* immediately before the intervention.

PCI	All patients		Patients with ≤90 sick-leave days	
	Women	Men	Women	Men
	*n = 6402*	*n = 26770*	*n = 3983*	*n = 18648*
	*PR (95% CI)* 1	*PR (95% CI)* 1	*PR (95% CI)* 1	*PR (95% CI)* 1
*Age groups (years)*				
30–49	1	1	1	1
50–54	**0.92 (0.86–0.98)** *	0.99 (0.95–1.04)	**0.88 (0.79–0.97)** *	0.95 (0.89–1.00)
55–59	0.95 (0.89–1.01)	1.02 (0.98–1.07)	0.93 (0.85–1.03)	0.98 (0.93–1.03)
60–63	**0.88 (0.82–0.94)** ***	0.99 (0.94–1.03)	0.90 (0.81–1.00)*	0.98 (0.92–1.04)
Women *vs* Men	**1.19 (1.16–1.23)** ***		**1.30 (1.25–1.36)** ***	
*Indication*				
MI^2^ or acute coronary syndrome				
*vs* stable angina	**1.29 (1.22–1.36)** ***	**1.30 (1.26–1.35)** ***	**2.08 (1.86–2.33)** ***	**2.32 (2.17–2.48)** ***
Diabetes mellitus	1.02 (0.96–1.08)	1.03 (0.99–1.08)	0.98 (0.88–1.09)	1.00 (0.94–1.06)
*Hospitalisation 5 years before the intervention*				
Cardiovascular disease^3^	1.05 (0.99–1.10)	1.04 (1.00–1.07)*	**1.17 (1.08–1.28)** ***	1.05 (1.00–1.10)
Mental disorders	1.06 (0.95–1.18)	1.03 (0.96–1.10)	1.13 (0.90–1.41)	0.99 (0.87–1.12)
Musculoskeletal disorders	0.99 (0.90–1.10)	1.03 (0.97–1.10)	**0.71 (0.54–0.95)** *	0.91 (0.80–1.03)
*Socio-economic classification*				
Higher non-manual employees^4^	1	1	1	1
Assistant non-manual employees	0.97 (0.90–1.05)	**1.20 (1.13–1.27)** ***	0.93 (0.83–1.04)	**1.14 (1.06–1.24)** **
Manual workers	1.04 (0.98–1.11)	**1.26 (1.21–1.31)** ***	1.04 (0.95–1.14)	**1.27 (1.20–1.33)** ***
*Employment status*				
Employed	1	1	1	1
Self employed	**0.50 (0.40–0.63)** ***	**0.86 (0.80–0.91)** ***	**0.41 (0.30–0.57)** ***	**0.62 (0.57–0.68)** ***
Unemployed	**0.41 (0.38–0.46)** ***	**0.75 (0.72–0.80)** ***	**0.35 (0.30–0.40)** ***	**0.34 (0.32–0.37)** ***
*Sick-leave days within one year before PCI*				
>90 *vs* 0–90 sick-leave days^5^	**1.90 (1.81–1.99)** ***	**2.33 (2.64–2.40)** ***	1.05 (0.92–1.20)	**1.21 (1.11–1.32)** ***

1 Adjusted for age and >90 sick-leave days *in total* during the year before PCI.

2 Myocardial infarction.

3 Acute myocardial infarction, heart failure, cerebrovascular disease and transient cerebral ischeamic attacks.

4 Including non-manual employees at intermediate level.

5 Not adjusted for >90 sick-leave days *in total* during the year before PCI.

* p-value <0.05;

** p-value <0.01;

*** p-value <0.001.

#### Statistical analyses

Mean and median number of sick-leave days in the first sick-leave spell following the cardiac intervention is presented, using date of the intervention to calculate sick-leave days before and after. To compensate for that data about sick-leave spells ≤14 days was not available for most patients, those with no registered sick-leave days following the intervention have been assigned 14 sick-leave days presented as weighted median. All analyses were done separately for women and men.

Due to a small number of female CABG patients in some years, the median number of sick-leave days after the intervention was computed for two years combined (moving median) in the figure. Thus, the median number of sick-leave days in 1995 is represented by the median number of days for the two years 1994 and 1995; for 1996 it is the median number of 1995 and 1996, and so forth.

In analyses of sickness absence after the intervention, patients with old-age pension (n = 1 106), disability pension >50% at the time of intervention (n = 11 331), patients who died (n = 346), or emigrated during the year after the intervention (n = 25) were excluded. Altogether, the analyses included 51 068 patients (17 896 CABG and 33 172 PCI).

Since mean and median number of sick-leave days following the two interventions differ, the following two definitions of the outcome variable *long-term sickness absence* have been used: >180 sick-leave days in the first sick-leave spell following CABG and >90 sick-leave days in the first sick-leave spell following PCI.

Our analyses are based on prevalent cases of the first long-term sick-leave spell following CABG or PCI. The prevalence ratio (PR) with 95% confidence intervals (CI) for long-term sickness absence adjusted for age and >90 sick-leave days in total the year before the intervention were calculated using a log-binomial function estimated by Generalized Linear Models (GLM) (PASW Statistics, version 19).

PR of long-term sickness absence adjusted for age, indication for intervention, diabetes, history of cardiovascular disease, socio economic position, and >90 sick-leave days in total the year before the intervention, was computed for the different periods of intervention with the period 1994–1996 as the reference.

**Table 5:** Prevalence ratio of a sick-leave spell >180 days following CABG for *all patients* and for *patients with ≤90 sick-leave days* immediately before the intervention.

	All patients		Patients with ≤90 sick-leave days	
	Women	Men	Women	Men
	*n = 2426*	*n = 15470*	*n = 1249*	*n = 9577*
	*PR (95% CI)* 1	*PR (95% CI)* 1	*PR (95% CI)* 1	*PR (95% CI)* 1
*Period of intervention*				
1994–1996	1	1	1	**1**
1997–2000	**1.20 (1.07–1.34)** **	**1.26 (1.18–1.34)** ***	1.22 (0.99–1.50)	**1.37 (1.23–1.53)** ***
2001–2003	**1.21 (1.08–1.36)** **	**1.32 (1.24–1.40)** ***	**1.39 (1.13–1.71)** **	**1.53 (1.37–1.71)** ***
2004–2006	1.13 (0.99–1.30)	**1.17 (1.08–1.26)** ***	1.24 (0.98–1.56)	**1.23 (1.09–1.38)** **

1 Adjusted for age, indication, diabetes, history of cardiovascular disease, socio economic position, and >90 sick-leave days *in total* the year prior to CABG.

* p-value <0.05;

** p-value <0.01;

*** p-value <0.001.

**Table 6:** Prevalence ratio of a sick-leave spell >90 days following PCI for *all patients* and for *patients with ≤90 sick-leave days* immediately before the intervention.

	All patients		Patients with ≤90 sick-leave days	
	Women	Men	Women	Men
	*n = 6402*	*n = 26770*	*n = 3983*	*n = 18648*
	*PR (95% CI)* 1	*PR (95% CI)* 1	*PR (95% CI)* 1	*PR (95% CI)* 1
*Period of intervention*				
1994–1996	1	1	1	**1**
1997–2000	1.04 (0.93–1.15)	1.06 (0.99–1.14)	1.01 (0.84–1.21)	1.11 (0.99–1.21)
2001–2003	1.06 (0.95–1.17)	**1.11 (1.03–1.18)** **	0.97 (0.81–1.16)	**1.15 (1.02–1.29)** *
2004–2006	0.96 (0.86–1.08)	0.98 (0.91–1.05)	0.89 (0.74–1.07)	0.95 (0.84–1.07)

1 Adjusted for age, indication, diabetes, history of cardiovascular disease, socio economic position, and >90 sick-leave days *in total* the year prior to PCI.

* p-value <0.05;

** p-value <0.01;

*** p-value <0.001.

## Results

In 2006, about 1000 men and 200 women of working age underwent CABG in Sweden. This was a reduction of more than 50% compared to in 1994 (Figure 1). During the same year, about 4000 men and 1000 women had a PCI, corresponding to a three-fold increase.

A majority of the patients were men; 84% and 78% among the CABG and PCI patients, respectively. For more than 50% of all patients, the main indication for both types of intervention was acute coronary syndrome (Table 1). About 70% of CABG patients and 30% of PCI patients had been hospitalised within five years before the intervention due to angina pectoris. A large proportion of the patients had hospitalisation for acute myocardial infarction, and diabetes mellitus was also common before the intervention. About half of all patients had a known employment or were self employed the year before the intervention, somewhat lower in women than in men. At the time of intervention, 40% and 35% of women in the CABG and PCI group were on disability pension, about two-fold compared to men. Most of the patients had some sickness absence the year before the intervention.

### Sickness Absence Following the Intervention

In the CABG group, 34% among women and 24% among men had at least 365 sick-leave days directly following the intervention (Table 2). Corresponding figures for the PCI patients were 24% and 15%. The median number of sick-leave days was 253 and 151 in women and men undergoing CABG and 71 and 47 in women and men undergoing PCI.

The median number of sick-leave days increased from 1997 until 2001 and thereafter decreased, with a slight increase in 2004 for female CABG patients (Figure 2). A similar pattern was found for the proportion of patients with at least 365 sick-leave days following CABG and PCI (Figure 3).

#### Risk of long-term sickness absence following CAGB

The prevalence ratio of having long-term sickness absence (>180 sick-leave days) immediately following CABG was 23% higher among women compared with men, after adjustment for age and long-term sickness absence during the year before the intervention (Table 3). Among women, older age, being self employed or unemployed was associated with a lower risk of long-term sickness absence. Among men, previous cardiovascular disease and diabetes, and being manual worker or assistant non-manual employee increased the risk of long-term sickness absence. The strongest factor for an increased risk for long-term sickness absence in both genders was a history of long-term sickness absence during the year before the intervention.

Essentially similar results was found in a separate analyses of individuals who had ≤90 sick-leave days immediately before the intervention (Table 3).

#### Risk of long-term sickness absence following PCI

Almost the same factors predicted long-term sickness absence (>90 sick-leave days) after PCI as for CABG (Table 4). However, self employed and unemployed in both genders had a lower risk of long-term sickness absence. Further, myocardial infarction or acute coronary syndrome as indication for intervention more than doubled the risk for long-term sickness absence among the patients with <90 sick-leave days immediately before the intervention.

#### Time trends in the risk of long-term sickness absence

Taking patient characteristics into account, there was an increase in the proportion of long-term sickness absence among CABG patients during the period 1994–2006 (Table 5). In the period 2004–2006 this increase was 13% among women and 17% among men compared to 1994–1996. Similar results were seen in patients with limited sickness absence before the intervention. In PCI patients, the proportion of long-term sickness absence following the intervention was stable when patient characteristics were taken into account (Table 6).

## Discussion

In this study we followed all patients of working age who underwent a first CABG or PCI in Sweden during the 13-year period 1994–2006, regarding sickness absence after the intervention. The results show that long-term sickness absence was very common, in particular among women. Furthermore, there was essentially no change in PCI patients and an increase in CABG patients regarding long-term sickness absence following the intervention when changes in patient characteristics were taken into account.

During the study period, there was a substantial reduction in the number of CABG patients and a dramatic increase in the number of PCI male patients in Sweden [12]. At the end of the study period, the patients undergoing CABG had more severe disease and more co-morbidity than corresponding patients at the beginning of the study period [1]. Among patients undergoing PCI, there were more acute medical cases at the end of the study period than at the beginning as PCI has become the preferred treatment option for patients with acute ST-elevation myocardial infarction [1]–[2]. This change in the indications for intervention for both the CABG and the PCI groups during the study period with more serious illness than in the beginning of the study period, may have influenced levels of sickness absence. There is no detailed information on extent of coronary artery disease in the registers, but we used the number of coronary bypass grafts sutured and number of stents per patients used at PCI as an indirect measure of this. As this was not associated with long-term sickness absence we did not include that in the analyses. We adjusted for several health factors, including previous sickness absence and morbidity. However, not all changes in patient characteristics were adjusted for in this study, e.g. we were not able to adjust for more effective medications and markedly reduced use of tobacco during the last decade. Residual confounding may have masked some reduction in long-term sickness absence due to an increased proportion of patients with more severe disease.

Compared to earlier studies, we found a substantially higher number of sick-leave days following coronary revascularisation [3]–[8], [10], [19]. Among the CABG patients, 34% among the women and 24% among the men were sickness absent for at least 365 days after the intervention and for the PCI patients the corresponding figures were 24 and 14%, respectively. Our higher figures could have different explanations. One is that previous studies were based on self-reported information on return to work [3]–[8], [10], [19]–[20], that older studies included patients with less severe disease, the relatively high employment rate among both women and men and in higher ages in Sweden [21], and the high retirement age [22]. Other explanations might be differences in sickness insurance systems, the system being very generous in Sweden and covering almost all people living in Sweden, also e.g. unemployed [17], [23]–[24].

Women had an increased risk compared to men of long-term sickness absence following both CABG and PCI. In Sweden, as in many other countries, women in general have higher sick-leave rates [17], [23]. To our knowledge, there are no previous studies in Sweden that have presented data about sickness absence following coronary revascularisation among both women and men. In a small Swedish rehabilitation study of patients with coronary artery disease, including CABG and PCI, it was more common with depression among women compared with men as well as an association between depression and no return to work [25]. The authors discussed that it might be more acceptable that women do not return to full-time work after coronary artery disease. In Sweden, as in many other countries women generally have a higher morbidity illustrated by higher drug consumption, higher hospitalisation and self reported ill-health compared with men as well as higher levels of sickness absence, irrespective of diagnoses [24]. The gender-segregated labor market means that women and men have different conditions and different physical and psychosocial risk factors in working life [26]–[27]. Also differences in cardiac rehabilitation, behaviour, lifestyle, and living conditions can be of importance [17]. These kind of data was not available in this study. However, no earlier studies have entirely explained the differences in sickness absence between women and men by these factors.

Long-term sickness absence prior to the intervention was a strong predictor of long-term sickness absence following the intervention, which is consistent with previous studies [9], [28]. In addition, no sickness absence or only short sick-leave spells before the intervention was more common among the PCI patients compared with the CABG patients and more common among men than among women. The higher proportion of patients without sick leave or only short sick-leave spells before the intervention from 1994 to 2006 could be explained by shorter waiting time before the intervention as well as an increased proportion of acute cases in particular among patients undergoing PCI [1]–[2].

Among women, more than 35% were on disability pension already at the time of the intervention. This high level of disability pension before coronary revascularisation among women has to our knowledge not been reported before. One possible explanation could be that cardiovascular disease commence later among women than among men and that women maybe had more co-morbidity and more severe illnesses compared with men. There are few studies on disability pension and even fewer of disability pension in relation to specific diagnoses [17].

Our results, like others, indicate that type of job is an important predictor of sickness absence following CABG as well as PCI [3], [5], [10], [19], [28]. Among men, a higher risk of long-term sickness absence was found among manual workers and non-manual employees compared to non-manual employees at intermediate or higher level. This was not seen among women. Obviously, the need for extended sickness absence varies depending on the type of job due to differences in physical and psychosocial demands [17]–[18]. The low risk for long-term sickness absence among self employed and unemployed is more difficult to explain in this way. A possible explanation is that self employed might feel that they cannot afford to be on long-term sick-leave or that they can adjust their work situation in a better way. That they had different number of qualifying days should not have affected the results – also the days with no benefits were recorded. This is an observation that deserves further investigation.

It remains to be evaluated to what extent the levels of sick-leave in this study corresponds to an optimal use of sickness absence after these interventions, considering the work incapacity of the patient. Notably, the national guidelines for sickness certification introduced in Sweden in 2007, recommends 2–4 weeks sickness absence after PCI and 2–3 months after CABG. This is considerably lower than the observed median numbers of sick-leave days in the present study. Of course, depending on the complications and type of work, impaired work capacity could remain for a long time. However, for most people work is a very important aspect for their quality of life and more knowledge about gender specific factors promoting and hindering return to work is warranted.

### Strengths and Limitations

The possibility to merge information from several national high-quality registers over a long-time period on an individual level for a very large population-based cohort and to take several potential confounding factors into account in the analyses makes this study unique. Another strength was that all persons living or working in Sweden were covered by a sickness insurance, principally providing free health care, that is, there was no bias in who underwent the interventions, based on economic situation. Moreover, essentially all PCI and CABG patients in working ages were included in the registers and thus, there was no selection of patients. The large cohort also made it possible to do separate analyses for women and men which has not been done previously. Further, the employment rate is comparatively high in Sweden, also among women in older ages, which makes gender comparisons more meaningful.

We only had information about sick-leave days for sick-leave spells with a duration of at least 15 days. To take this into account we also present the weighted median number of sick-leave days. However, the total number of sick-leave days in this study is somewhat underestimated. Nevertheless, the duration of most sick-leave spells following the intervention was often so long that this has not much influenced the results.

Information about socioeconomic position was from 1990, and fairly old at the end of the study period. However, social class is a fairly stable characteristic and the misclassification of social class was most likely of limited importance.

### Conclusions

This unique national study covering a 13-year period shows that long-term sickness absence following coronary revascularisation is common in Sweden, especially among women, and is associated with socio-economic position, co-morbidity, and sickness absence during the year before the intervention. Gender specific scientific knowledge about use and effects of sickness absence following coronary revascularisation is warranted for the patients, the treating physicians, the healthcare sector, and the society.
